# Phytochemical Screening and Toxicological Study of* Aristolochia baetica* Linn Roots: Histopathological and Biochemical Evidence

**DOI:** 10.1155/2019/8203832

**Published:** 2019-02-03

**Authors:** Mohammed Bourhia, Amal Ait Haj Said, Ayoub Chaanoun, Fatiha El Gueddari, Abderrahim Naamane, Laila Benbacer, Naima Khlil

**Affiliations:** ^1^Laboratory of Chemistry-Biochemistry, Environment, Nutrition, and Health, Faculty of Medicine and Pharmacy of Casablanca, Hassan II University, 19 rue Tarik Ibn Ziad, Casablanca, Morocco; ^2^Laboratory of Pharmacognosy, Faculty of Medicine and Pharmacy of Casablanca, Hassan II University, 19 rue Tarik Ibn Ziad, Casablanca, Morocco; ^3^Laboratory of Health and Agri-Food, Faculty of Science and Technology, University Hassan I, Route de Casablanca Km. 3,5, BP 539, Settat, Morocco; ^4^Life Science Division, National Centre for Energy, Sciences, and Nuclear Techniques, BP 1382, 10001 Rabat, Morocco

## Abstract

*Aristolochia baetica *(A*. baetica*) is a wild species of Aristolochiaceae family; its roots are used by Moroccan people against cancer for many years ago. The objective of the study was to investigate the phytochemical screening, acute and subacute toxicity of* A. baetica* roots growing in the north of Morocco. Qualitative and quantitative analyses of* A. baetica* roots were performed using standard methods; the acute toxicity of the root extract of the studied plant was assessed in mice by gavage of single doses of 1, 2, and 4 g/kg body weight for 14 days; by the time the subacute toxicity was done using repeated doses 1, 1.5, and 2 g/kg/day for 28 days. Histological changes and biochemical parameters as markers of kidney and liver function were evaluated. The results of phytochemical screening showed the presence of polyphenols, tannins, alkaloids, flavonoids, saponins, and the absence of anthraquinones, sterols, and terpenes. The results of acute toxicity showed the absence of mortality and signs of toxicity in groups treated with 1 and 2 g/kg; however, the clinical signs of toxicity were important and the rate of mortality was estimated at 16 % in the group treated with 4 g/kg. The results of subacute toxicity showed several changes of serum parameters registered in groups treated with 1.5 and 2 g/kg/day, respectively. The results showed also the absence of histological injuries in groups treated with 1 and 1.5 g/kg/day; meanwhile, the histological alterations were remarkable in treated group with the highest dose administered of 2 g/kg/day. The outcome of this work showed that the roots' extract of the studied plant was toxic in mice with repeated doses, but no toxic effect was observed with a single dose under 4g/kg.

## 1. Introduction

For many years ago, the medicinal plants have been largely used in the treatment of many diseases throughout the world; plants contain naturally a large variety of chemical substances with different pharmacological and biological activities. As reported in the literature, the percentage of Moroccan people using traditional medicines ranges from 50 to 75 % [1]; meanwhile, many other studies have shown that a huge quantity of herbs which used without scientific proof may overexert toxic effects [2].

*A. baetica* belongs to the Aristolochiaceae family, is a wild species used by the Moroccans against several diseases since ancient time; especially the roots prepared in water are used against cancer [3], and digestive diseases [4], the aerial parts are utilized to treat abortifacient, the flower parts are used to treat rheumatic. The whole plant of* A. baetica* is also decocted in water and used as anti-inflammatory and antiseptic in many regions of Morocco [5]. As matter of fact the preparation including plants of genus Aristolochia is banned because of their toxicities due to aristolochic acids (AAs). The AAs case was detected at first in Belgium into a group of women patients who was affected by critical renal disease after ingesting the plant of* Aristolochia fangchi *for a long time [6]. Aristolochic acids have been recognized to be toxic for neurons [7], carcinogenic [8], and mutagenic [9]; the herbal remedies which contain the plant including genus Aristolochia have been banned in many countries throughout the world [10]. The responsible places of health agencies of Morocco declared in press that some cases of renal failures were registered due to adopting a preparation including Bereztem [11]; on the other hand some cases of death were recorded in Morocco due to using* A. longa *in the preparation against cancer [12].

It is kindly noted that the aim of this study was to evaluate the phytochemical screening, acute and subacute toxicity of* A. baetica* prepared in decoction; thus, different doses of the studied plant were administered to mice, biochemical parameters and histological changes were studied

## 2. Materials and Methods

### 2.1. Plant Material

Roots of* A. baetica *were harvested from Meknes region a city in Morocco about 150 km east of Moroccan capital (Rabat) in October 2016. A voucher specimen were taxonomically identified and deposited in the Herbarium of Scientific Institute of University Mohammed V–Rabat–Morocco.* A. baetica roots *were washed with water, dried at room temperature, and ground into a fine powder using an electric mixer.

### 2.2. Aqueous Extract Preparation

The aqueous extract of* A. baetica *was prepared following the standard traditional method described in the literature [13]. The powder of roots was first boiled for 20 min at 100°C. Thereafter, the mixture obtained was cooled at room temperature and then centrifuged, and the supernatant was filtered using Whatman filter paper. The filtrate was concentrated in a rotary vacuum evaporator. The crude extract reconstituted on a daily basis in distilled water for final concentrations required for oral administration [14].

### 2.3. Preliminary Qualitative Phytochemical Screening

The plant material was subjected to qualitative phytochemical screening in order to qualitatively determine some type of interested phytoorganic constituents which are responsible for biological activities, alkaloids, flavonoids, polyphenols, anthraquinones, saponins, tannins, sterols, and terpenes which were the major cheeked groups using standard methods.

### 2.4. Animal Material

Adult Swiss albino mice weighing approximately 25 g were used for both acute and subacute toxicity; the mice were purchased from the animal colony of Pasteur Institute (Casablanca, Morocco). All animals were kept in polypropylene cages. The animals were acclimatized for one week under laboratory conditions of regular light/dark cycles (12/12 h) and temperature (24 ± 2°C). The animals had free access to tap water and normal pellet diet [15].

### 2.5. Study of Acute Toxicity

A total of 24 male adult mice were randomly divided into 4 experimental groups of 6 mice each; the animals were grouped according to selected doses of plant extract, one control group and three treatments. After fasting overnight, the aqueous extract of the plant roots was administered to each treatment group at single doses of 1, 2, and 4 mg/kg body weight; by the time the control group received an equivalent volume of distilled water (vehicle). After treatment, the mice were observed individually for clinical symptoms, mortality, and changes in general behavior during the period of treatment [16]. This study was conducted according to the Organization for Economic Cooperation and Development (OECD) Guideline No. 425 [17].

### 2.6. Study of Subacute Toxicity

The subacute toxicity study was effectuated according to the Organization for Economic Cooperation and Development (OECD) Guideline No. 407 [18]. A total of 24 male adult mice were randomly segregated into 4 groups of 6 mice per group for each; the mice were grouped according to selected doses of plant extract chosen to be tested, one control group and three treatments. Animals in treatment groups received repeatedly* B. dioica* root extract at doses of 1, 1.5, and 2 mg/kg/day for 28 days; by the time the control group received an equivalent volume of distilled water (vehicle). During the treatment period, the weight of animals was measured once a week. Animals were also observed for signs of toxicity, mortalities, changes in general behavior, and changes in physical appearance [19].

### 2.7. Biochemical Examination

On the 28 the day, all surviving animals were deprived of food overnight and sacrificed for blood collection. Heparin Tubes containing collected blood sample were centrifuged at 6000 rpm at 4°C for 20 min in order to obtain the serum. The measurement was effectuated according to the method described by Taj et al. (2014) [20], using an automated analyzer (roche cobas mira plus, Switzerland). The clinical biochemistry parameters included aspartate aminotransferase (AST), alanine aminotransferase (ALT), lactate dehydrogenase (LDH), urea, and creatinine.

### 2.8. Histopathological Evaluation

At the end of the treatment period, the major organs (liver and kidney) were removed for histopathological examination. The organs were carefully fixed in 10 % buffered formalin (pH 7.4). After fixation, tissue specimens were dehydrated in a graded series of ethanol (70–100%). After organization cropping and paraffin embedding, tissue specimens were stained with Haematoxylin and Eosin (H&E) prior to microscopic examination. The pathological examination of liver and kidneys tissue was carried out by a pathologist using a light microscope. The microscopic features of examined organs of treated groups were compared to the control group [21].

### 2.9. Statistical Analysis

All quantitative data were expressed as the means ± SD (standard deviation). Statistical significance between the means of control and treated groups was determined by one-way ANOVA using GraphPad Prism 7 software. The means were pairwise compared using Tukey test and the differences were considered statistically significant at p less than 0.05.

## 3. Results

### 3.1. Qualitative Phytochemical Screening

The findings of preliminary qualitative phytochemical screening of* A. baetica * roots were shown in Table 1; they revealed the presence of flavonoids, polyphenols, alkaloids, tannins, and saponins and the absence of anthraquinone, sterols, and terpenes.

### 3.2. Acute Toxicity

During the acute toxicity study, no mortalities or signs of toxicity occurred in mice treated with a dose less than 4 g/kg; the mice were only characterized by an accelerated running about 3 to 5 min. However, the treatment with a dose of 4g/kg was responsible for shortness of breath, abnormal locomotion, hypoactivity, tending to deepen and be gentle, salivation, lack of appetite, lethargy, reversal reflection, occasional convulsion, and 16 % of deaths.

### 3.3. Subacute Toxicity

The general behavior of the mice and signs of toxicity were observed considering the mortality during the 28 days of feeding the aqueous extract; during the whole period of dosing, no visible toxic effects were noted in groups treated with 1 and 1.5 g/kg/day. From the 2nd week of treatment the mice treated with 2g/kg/day represented abnormal locomotion, ataxia, anorexia, hypoactivity and depression.

#### 3.3.1. Effect of Aqueous Extract on the Mice Weight during the Treatment Period

**Figure 1:**
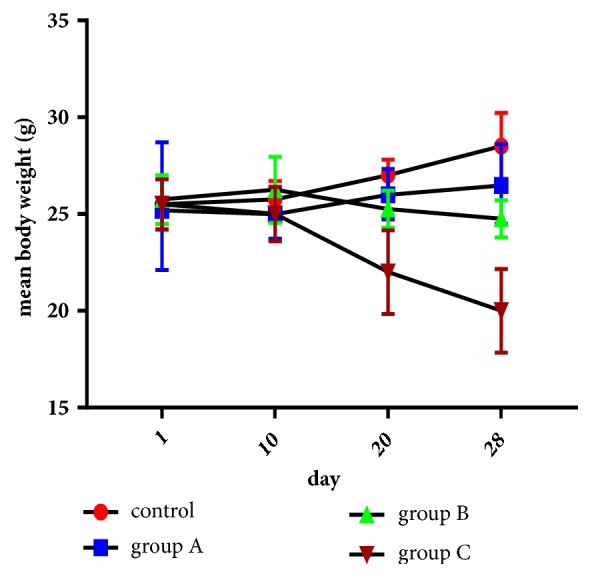
Changes of mice weight during the treatment period with doses 1 g/kg/day (group A), 1.5 g/kg/day (group B), and 2 g/kg/day (group C); results represent the means ± SD (standard deviation).

During the treatment period, we did not note a significant change in the weight of treated mice with 1 and 1.5 g/kg/day body weight (group A and B) compared to the control group (p>0.05). This slight variation in weight may be due to the nervousness and stress of animals during and after swabbing. On the other hand, the treated mice with dose of 2 g/kg/day (group C) caused a weight loss from the second week of treatment, which became significant after 20 and 28 days of treatment (p*∗*<0.05) (Figure 1).

#### 3.3.2. Effect of Aqueous Extract on Biochemical Parameters

**Figure 2:**
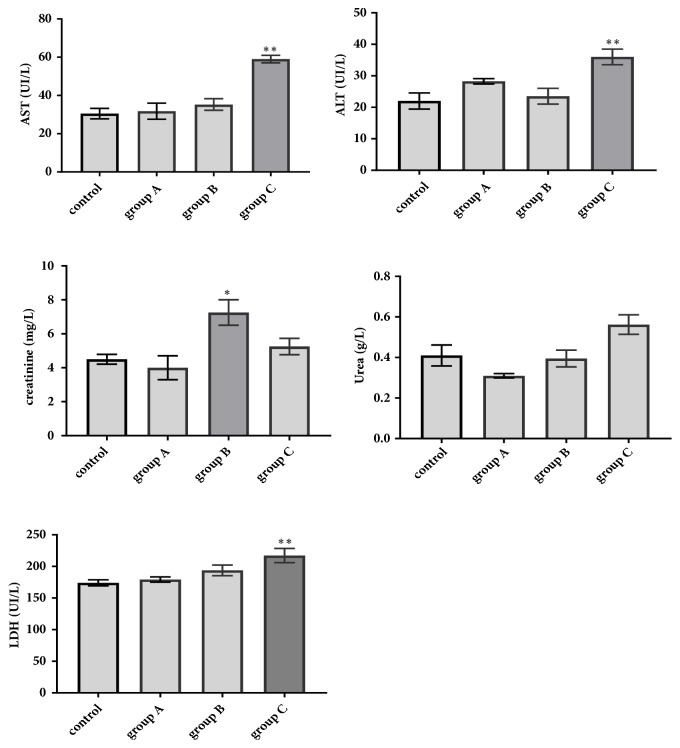
Effect of the plant extract at doses 1 g/kg/day (group A), 1.5 g/kg/day (group B), and 2 g/kg/day (group C) on the transaminases (ALT; AST), urea, creatinine, and lactate dehydrogenase (LDH) after the end of the experiment; results represent the means ± SD (standard deviation).

Some of the biochemical markers of liver function such as alanine aminotransferase (ALT), aspartate aminotransferase (AST), and kidney function such as urea, creatinine and lactate dehydrogenase (LDH) as a ubiquitous enzyme were measured. The results are represented in Figure 2.

Regarding the liver markers, the biochemical results showed a significant increase of AST dosed in group C (2 g/kg/day) compared to that recorded in the control group (p*∗∗*<0.05). However, there is no significant change observed in groups A and B treated, respectively, with 1 and 1.5 g/kg/day) (p>0.05). regarding ALT transaminases, there is insignificant increase registered in groups A and B treated, respectively, with 1 and 1.5 g/kg/day compared to the control group; meanwhile we note a significant increase in group C (2 g/kg/day) (p*∗∗* <0.05).

Regarding the renal markers, the creatinine concentration was increased in group B (1.5 g/kg/day) compared to the control group (p*∗*<0.05). Meanwhile, there is no significant increase noted in group A (1 g/kg/day) and C (2g/kg/day), considering urea, the results showed insignificant change in all treated groups compared to the control lot (p>0.05). In regard to ubiquitous enzyme (LDH), we did not register any significant increase in groups A and B treated with 1 g/kg/day and 1.5 g/kg/day, respectively, compared to the control group (p>0.05). However, a significant increase was observed in group C treated with 2 g/kg/day (p*∗∗*<0.05)

#### 3.3.3. Histopathological Changes

#### 3.3.4. Kidney

**Figure 3:**
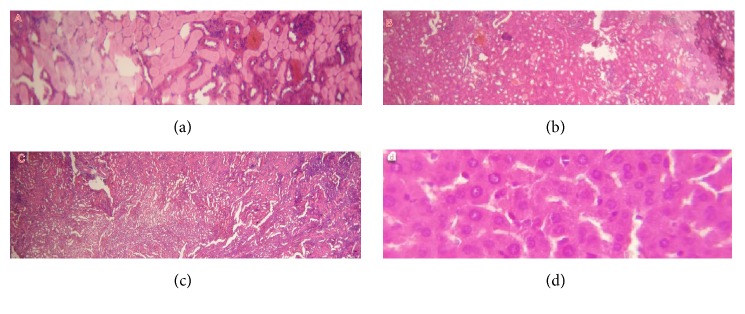
Histologic section of kidney tissue of control and treated mice (section of parenchyma stained with H&E, x 40). (a), (b), and (c) are histologic sections of kidney tissue of treated mice with 1.5 and 2 g/kg/day; (d) is a section of kidney tissue of control mice.

The histopathological examinations of the renal tissue showed that no histopathological changes occurred in the kidney of group A (1 g/kg/day); however, the renal tissue of groups treated with doses 1.5 and 2g/kg/day represented renal necrosis, inflammatory infiltrate, cortical necrosis, and tubular degeneration; the major results are summarized in Figure 3.

(A) Renal necrosis, (B) cortical necrosis, and (C) minimal inflammatory infiltrate.

#### 3.3.5. Liver

**Figure 4:**
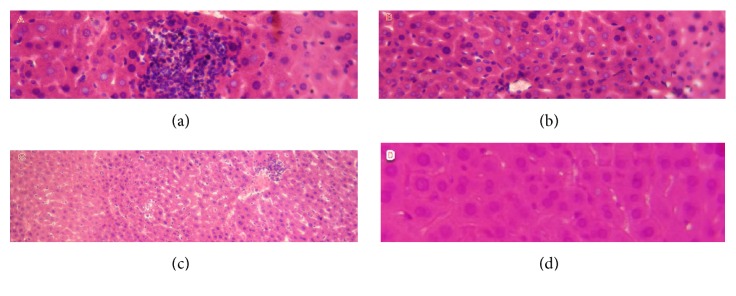
Histologic section of liver tissue of control and treated mice (section of parenchyma stained with H&E, x 40). (a), (b), and (c) are histologic sections of liver tissue of treated mice with 1.5 and 2 g/kg/day; (d) is a section of liver tissue of control mice.

The microscopic observation showed that no remarkable histopathological changes occurred in the liver of treated groups with doses of 1 and 1.5 g/kg/day; on the other hand, the renal tissue of group C (2g/kg/day) was described by inflammatory infiltrate, hepatic necrosis, and hepatic cholestasis; the major results are summarized in Figure 4.

(A) Inflammatory infiltrate and hepatic necrosis, (B) hepatic necrosis, and (C) minimal inflammatory infiltrate.

## 4. Discussion

Herbal medicines are largely appreciated by the public because it is believed to have no side effects due to their natural origins and are often seen as safe food supplements and not drugs. Medicinal plants are often self-prescribed by herbalist without control and review in terms of posology, manner, and frequency of administration. In reality, the chemicals in the medicinal plant may be naturalistic to the plant itself, but they are not naturalistic to the human body. Truly any chemical compound with therapeutic effect has a possibility of being erroneously prescribed or overdosed.

The phytochemical profile of the plant extract revealed that the presence of alkaloids, polyphenols, flavonoids, tannins, and saponins could be the responsible compounds for the toxic effect of the studied plant. According to results of acute toxicity the rate of mortality was estimated at 16 % due to highest dose feed (4g/kg); considering the scale of Viala (1998) [22], the aqueous extract of roots decoction could be not or very little toxic [23]. On the other hand, there is no correlation between LD_50_ and aristolochic acids content recorded in the acute toxicity of organic extract of* Aristolochia manshuriensis *as reported in the literature [23]. Hence, these results suggest that other components in the extract may be dominating the acute toxicity.

Under the conditions of acute and subacute toxicity of roots decoction of* A. baetica,* the clinical signs observed such as hypoactivity and weight loss may be related to lack of appetite [6], lethargy, salivation, and anorexia which could be attributed to properties of the studied plant [24]; the seizures listed showed us the probable neurotoxic effect of roots decoction of* A. baetica* [25]. Our findings were compared to those reported in the study of subacute toxicity of* A. fructus* [19], in which it was reported that the animals feed with 22.2 g/kg of plant extract of* A. fructus *were affected by phenomenon, convulsion, reversal reflection and shortness of breath, dim and rough hair, and the ptosis closing.

Considering the findings of biochemical parameters, increased plasmatic level of urea and creatinine indicated that the roots' decoction of* A. Baetica *proved to be toxic [26]. On plasma level, we also noticed an increase in LDH (a soluble enzyme found normally inside every living cell) level into the surrounding extracellular space compared to the control mice, and this put us to think about the cell damage effects of roots decoction of* A. baetica *[27]. These obtained findings were supported by others reported in the toxicity studies conducted by Cherif and his collaborators, in which it was reported that the administration of aqueous extract of* Aristolochia longa* roots for 28 days affects the biochemical parameters in mice with a dose of 2.5 g/kg/day body weight [28]. The biochemical findings (disturbance of biochemical parameters) are confirmed by those obtained from histological examination of kidney and liver. Moreover, our results were compared to those discussed in the chronic toxicity of* A. manshuriensis *in which the authors reported that the absence of any significant biochemical variation after feeding mice with organic extract for eight weeks [14–23]. Thus, the organic extract of* A. manshuriensis *was responsible for hepatic necrosis [19]. Many studies showed that the oral administration of 2.5g/kg/day of the aqueous extract of* A. longa* induces histological injuries in the liver and kidneys, such as altered tissue architecture, lymphocytic infiltrates, foci of hemorrhage, hepatic-cholestasis, tubular necrosis, and cell congestion. Our results of histopathological changes such as renal necrosis, cortical necrosis, inflammatory infiltrate, and hepatic necrosis were supported by those obtained from biochemical evaluation; these findings were similar to those reported in the study of Cherif et al. [27–29]. Furthermore, our results are also comparable to those reported in the literature in which it was stated that the subacute toxicity of aqueous extracts of* Aristolochia Fructus* revealed a dose-dependent relationship of nephrotoxicity. However, the safe clinical dose of* A. Fructus* was 3.0 g/day for adults [19].

Taking into account the abundance of inflammatory infiltrate in two examined organs and especially into surrounding lesions, the histopathological results recorded during this work could be attributed to immunomodulatory properties of roots extract of* A. baetica* which might trigger an autoimmune response in the toxic lesions [29]. In point of fact, several studies have shown that ingestion of medicinal plants including genus Aristolochia could be responsible for severe renal injury, including renal interstitial fibrosis [30, 31]. It should be noted that* A. baetica *and 31 related species are recognized to contain Aristolochic acids [5].

## 5. Conclusion

According to results of acute toxicity reported in this work, the aqueous extract of* A. baetica* was little toxic; meanwhile, the same extract showed high toxicity on serum parameters and histologic tissues when ingested for a long time under the subacute toxicity conditions. Considering our outcome of this study, we need to pay more attentions to the biodiversity of plants for safety control

## Figures and Tables

**Table 1:** Phytochemical screening of *A. baetica *roots.

Polyphenols	+++
Alkaloids	+++
Flavonoids	+++
Anthraquinone	-
sterols and terpenes	-
saponins	++
Tannins	+++

+++: strong positive test; ++ positive test; +: low positive test; *–*: negative test.

## Data Availability

(1) Previously reported “Immunostimulatory Potential of* Aristolochia longa* L. Induced Toxicity on Liver, Intestine and Kidney in Mice” data were used to support this study and are available at A Histopathological analyses of in vivo antitumor effect of an aqueous extract of Aristolochia longa used in cancer treatment in traditional medicine in Morocco', Int J Plant Res, vol. 2, pp. 31–35, 2012. These prior studies (and datasets) are cited at relevant places within the text as reference [29]. (2) Previously reported “Late Onset of Bladder Urothelial Carcinoma after Kidney Transplantation for End-Stage Aristolochic Acid Nephropathy” data were used to support this study and are available at 10.1053/j.ajkd.2007.11.015. These prior studies (and datasets) are cited at relevant places within the text as reference [7]. (3) Previously reported “Aristolochic Acid Induces Proximal Tubule Apoptosis and Epithelial to Mesenchymal Transformation” data were used to support this study and are available at 10.1038/sj.ki.5002714. These prior studies (and datasets) are cited at relevant places within the text as reference [12]. (4) Previously reported “Acute Toxicity Evaluation of Ethanolic Extract of* Aristolochia albida* Duch. Leaves on Wistar Rats Liver and Kidney Functions” data were used to support this study and are available at 10.22159/ijpps.2017v9i7.16887. These prior studies (and datasets) are cited at relevant places within the text as reference [19]. (5) Previously reported “Studies on the Toxicity of Aristolochia manshuriensis (Guanmuton)” data were used to support this study are available at DOI: 10.1016/j.tox.2004.01.026. These prior studies (and datasets) are cited at relevant places within the text as reference [23]. (6) Previously reported “Toxic Effects of Some Medicinal Plants Used in Moroccan Traditional Medicine” data were used to support this study and are available at Moroc. J Biol, vol. 2, no. 3, pp. 21–30, 2006. These prior studies (and datasets) are cited at relevant places within the text as reference [1].
